# Tracheostomy-related indications, early complications and their predictors among patients in low resource settings: a prospective cohort study in the pre-COVID-19 era

**DOI:** 10.1186/s12893-023-01960-5

**Published:** 2023-03-18

**Authors:** Daniel J. Nyanzi, Daniel Atwine, Ronald Kamoga, Caroline Birungi, Caroline A. Nansubuga, Victoria Nyaiteera, Doreen Nakku

**Affiliations:** 1 Department of Otolaryngology, School of Medicine, Kabale University, Kabale, Uganda; 2 Department of Clinical Research, SOAR Research Foundation, Mbarara, Uganda; 3 Department of Otolaryngology, Mbarara University of Science and Technology, Mbarara, Uganda; 4 Department of Anatomy, Mbarara University of Science and Technology, Mbarara, Uganda; 5 Department of Pediatrics and Child Health, Makerere University College of Health Sciences, Kampala, Uganda

**Keywords:** Tracheostomy, Indication, Incidence, Early complications, Pre-Covid-19

## Abstract

**Background:**

Tracheostomy is a life-saving procedure whose outcomes may vary between hospitals based on disparities in their existing expertise. We aimed at establishing the indications, early tracheostomy-related complications and their associated factors in Uganda.

**Methods:**

In a prospective cohort study, we consecutively enrolled one-hundred patients, both adults and children 2 h post-tracheostomy procedure. At baseline, information on patients’ socio-demographics, tracheostomy indications, pre- and post-procedural characteristics was collected through researcher administered questionnaires and from medical records. Clinical examination was performed at baseline but also at either day 7 or whenever a tracheostomy-related complication was suspected during the 7 days follow-up. Comparison of patients’ baseline characteristics, tracheostomy indications and complications across two hospitals was done using Pearson’s chi-square. For predictors of early tracheostomy complications, bivariate and multivariate analysis models were fitted using binomial regression in STATA 13.0 software.

**Results:**

All patients underwent surgical tracheostomy. Majority were adults (84%) and males (70%). The commonest tracheostomy indications were; pulmonary toilet (58%) and anticipated prolonged intubation (42%). Overall, 53% (95% CI: 43.0 – 62.7) had early complications with the commonest being tube obstruction (52.6%). Independent predictors of early tracheostomy-related complications were; anticipated prolonged intubation as an indication (RR = 1.8, 95%CI: 1.19 – 2.76), Bjork flap tracheal incision (RR = 1.6, 95%CI: 1.09 – 2.43), vertical tracheal incision (RR = 1.53, 95%CI: 1.02 – 2.27), and age below 18 years (RR = 1.22, 95%CI: 1.00 – 1.47).

**Conclusion:**

Pulmonary toilet is the commonest tracheostomy indication at major hospitals in Uganda. The incidence of early tracheostomy complications is high and majorly related to post-procedure tracheostomy tube management. Having anticipated prolonged intubation as an indication for tracheostomy, a Bjork flap or vertical tracheal incisions and being a child were associated with increased risk of complications. Emphasis on multidisciplinary team care, standardization of tracheostomy care protocols, and continuous collection of patient data as well as paying attention to patient quality of life factors such as early return to oral feeding, ambulation and normal speech may have great potential for improved quality of tracheostomy care in low resource settings.

## Background

Tracheostomy is a life-saving surgical procedure used to secure the airway. There are a number of indications for tracheostomy but the common ones include acute respiratory failure, anticipated need for prolonged mechanical ventilation, broncho-pulmonary toilet, and impacted airway foreign bodies among others [1, 2]. In the intensive care unit (ICU), the procedure has been noted to reduce the requirements for patient sedation when prolonged intubation is anticipated, improve patient comfort especially if ventilated, and facilitate earlier resumption of patient respiratory autonomy [2, 3].

The many benefits of tracheostomy have led to a global upward trend in the frequency of the procedure with up-to 250,000 procedures currently performed annually in resource-rich countries [4] and indications are being continuously revised. In literature, this rising trend has been attributed to increased access to better intensive care services [5] and changes in the epidemiology of upper airway obstructive conditions [6]. In the past, obstructive airway disease secondary to acute aero-digestive infections was the most common indication, but in the recent pre-COVID-19 years, trauma and aero-digestive tumors have dominated the list [6–8]. It is important to note that some special circumstances may require reconsideration of the indications and timing for tracheotomy surgery for example, during the COVID-19 pandemic, several centres adjusted quickly with regard to how tracheostomies were done among critically ill patients in lieu of protection of health workers from the highly infectious disease. This emphasizes the need for flexibility in guidelines for tracheostomy surgery depending on the case, surrounding circumstances and benefit of the procedure [9, 10].

Like all surgical procedures, tracheostomy carries a risk of adverse events that may increase patient morbidity, prolong hospital stay and add undue strain to an already low resourced healthcare system [2, 7, 11]. Common complications of tracheostomy include stomal site infection, bleeding, tube obstruction and inadvertent tube dislodgement among others. Even though literature suggests that tracheostomy related complication rates range from 6 to 66% with a mortality rate of up-to 60% depending on existing comorbidities [4, 12, 13], it is unclear if this can be generalized to resource limited settings. Furthermore, differences in hospital resources and policies may play a contributory role to variation in patient outcomes and complication rates. For example, in high resource centers, different cadre of staff ranging from surgical residents, anesthesiologists, surgeons and critical care nurses may be trained to perform this complex procedure and care for the patients thereafter. However, in low resource settings like Uganda, it is reserved for specially trained and in-training medical personnel such as otolaryngologists and oromaxillofacial surgeons while post-procedural care is offered by the nurses and patient attendants. We hypothesize that this may contribute to variation in complication rates for tracheostomy. Other factors that have been documented in literature to be associated with tracheostomy-related complications include; obesity, patient age and tracheostomy type among others [7, 14–16]. Unfortunately, there is scanty literature on the incidence of tracheostomy-related complications in Uganda.

Therefore, our study aimed at establishing the indications for tracheostomy, the incidence of early tracheostomy-related complications and their associated factors in Uganda.

## Methods

We conducted a prospective cohort study of adult and child patients who had under-gone tracheostomy at least 2 h prior to recruitment. A patient was excluded from participation if he or she had: a history of a known bleeding disorder, had no caretaker in hospital, and if they had had the tracheostomy performed from other hospitals besides those involved in this study. In order to achieve the sample size, recruitment was performed at two large university training hospitals in Uganda, that is, Mbarara Regional Referral Hospital (MRRH) in South western Uganda, and Mulago National Referral Hospital (MNRH) in the capital city of Uganda. In both hospitals, the Otolaryngology division is solely tasked with performing tracheotomy procedures.

The sample size of 100 patients was calculated using the formula for estimation of a single population proportion, that is; *n* = Z^2^*P (1—P)/r^2^ [17], where: Z = standard normal deviation for two-tailed test based on alpha level (relates to the confidence interval level), assumed at 95% = 1.96; P is the proportion of patients with tracheostomy-related complications, assumed to be 0.42, based on a 42% prevalence of tracheostomy related complications reported in a study in Kenya [14]; r is the margin error of estimation that was assumed to be 0.1, that is 10%, plus a 6% addition in sample size to cater for attrition. Ethics approval and consent to participate was received from the Mbarara University of Science and Technology Research Ethics Committee under approval number MUST-REC 01/11–17 prior to commencement of study activities.

Written informed consent of eligible patients was obtained prior to enrolment into the study. At both sites, patients were consecutively recruited based on the eligibility criteria two hours post-procedure from different units likely to host tracheotomy patients including the intensive care and high dependence units, emergency units and surgical wards. The 2-h period was anticipated to be adequate for a patient’s post-anesthetic stability and safe transfer from the procedure room to the host ward for post-operative monitoring and care. The decision was further guided by reports that tube obstruction, which is the most anticipated early complication is more likely to occur after 2 h post-procedure [15]. For patients who were not fully conscious at the time of enrolment, consent was sought from their attendants.

Patients’ socio-demographics, indications of tracheostomy, pre- and post-procedural factors including complications were collected at baseline from the patients, their medical records and caretakers using a researcher administered questionnaire. A baseline and a day 7 clinical examination was performed so as to establish the complications of tracheostomy. In addition, clinical examination was performed whenever a complication was suspected during the 7 days of follow-up. As a quality control measure, the questionnaire was pretested before its use in the study. The principal investigator also trained the research assistants who were ICU nurses and otolaryngology residents on the study procedures especially how to recognize the tracheostomy-related complications.

Data was analyzed using STATA version 13.0. Patients’ baseline characteristics were described using medians for continuous variables and proportions for categorical variables. Stratified analysis was conducted to compare the frequency of each indication across hospitals using Pearson chi-square test. Complications were reported as frequencies and a composite variable of tracheostomy-related complication was generated as a binary variable coded 0 = “No early tracheostomy-related complication”, and 1 = “early Tracheostomy-related complication”. An individual was considered to have an early tracheostomy-related complication if he/she had at-least one of the pre-defined complications both at enrolment and over the 7 days of study follow up. The overall proportion of patients with at-least one early tracheostomy related complication was calculated as the number of patients who developed at least one complication out of the total number of patients enrolled and expressed as a percentage, while the incidences of specific complications were calculated as frequencies of specific complications out of the total cumulative number of complication events and expressed as percentages. For complications such as tube obstruction which occurred more than once in some patients, only one event was recorded per patient and the total number of reported such events for all patients constituted the total cumulative number of complications.

Using chi-square and binomial regression models in univariate and multivariate analysis, the factors associated with early tracheostomy-related complications during follow-up were established. A significance level of 5% was used. Both unadjusted and adjusted risk-ratios were presented in tables with their corresponding 95% Confidence Intervals.

## Results

A total of 100 patients who had all undergone surgical tracheostomy were recruited into the study over a period of one year from MNRH and MRRH. Majority of these were male (70%) and adult patients (84%). Majority of the tracheostomies were performed in ICU/HDU settings (54%), and were emergency tracheostomies in 43% of patients. Peri-operative antibiotics were administered in 60% of patients Table 1.

**Table 1:** Sociodemographic and peri-surgical characteristics of patients, overall and by Hospital stratification

Characteristic	Overall, *N* = 100, n (%)	MNRH, *N* = 50, n (%)	MRRH, *N* = 50, n (%)	*p*-value
Gender				
Male	70 (70.0)	33 (66.0)	37 (74.0)	0.383
Female	30 (30.0)	17 (34.0)	13 (26.0)	
Age (Years), Median (IQR)	37 (25–59)	30 (22–58)	39.5 (29–65)	
Age categories (Years)				
Adults (≥ 18)	84 (84.0)	39 (78.0)	45 (90.0)	0.102
Pediatric (< 18)	16 (16.0)	11 (22.0)	5 (10.0)	
Family Socioeconomic class^*^				
Upper middle	7 (7.1)	2 (4.1)	5 (10.2)	0.058
Lower middle	23 (23.5)	17 (34.7)	6 (12.2)	
Upper lower	54 (54.1)	24 (49.0)	30 (61.2)	
Lower	14 (14.3)	6 (12.2)	8 (16.4)	
Admission diagnosis				
TBI/stroke	60 (60.0)	28 (56.0)	32 (64.0)	0.591
Upper airway obstruction	34 (34.0)	18 (36.0)	16 (32.0)	
Others	6 (6.0)	4 (8.0)	2 (4.0)	
Site for surgery				
Emergency/Casualty	7 (7.0)	6 (12.0)	1 (2.0)	0.000
ICU/HDU	54 (54.0)	22 (44.0)	32 (64.0)	
Operating theatre	25 (25.0)	8 (16.0)	17 (34.0)	
Wards (ENT/OMF)	14 (14.0)	14 (28.0)	0 (0.0)	
Type of tracheostomy				
Elective	57 (57.0)	25 (50.0)	32 (64.0)	0.157
Emergency	43 (43.0)	25 (50.0)	18 (36.0)	
Type of tracheal incision				
T-incision	39 (39.0)	2 (4.0)	37 (74.0)	0.000
Bjork flap	9 (9.0)	0 (0.0)	9 (18.0)	
Vertical^*^	48 (48.0)	47 (94.0)	1 (2.0)	
Horizontal	4 (4.0)	1 (2.0)	3 (6.0)	
Closure of skin surgical incision^*^	71 (71.7)	25 (50.0)	46 (94.0)	0.000
Cuffed tracheostomy tube use	89 (89.0)	47 (94.0)	42 (84.0)	0.110
Disposable internal cannula use	28 (28.0)	3 (6.0)	25 (50.0)	0.000
Peri-operative antibiotic use	60 (60.0)	24 (48.0)	36 (72.0)	0.014

Family socioeconomic classification based on the 2018 modified Kuppuswamy Socioeconomic Scale [18]

^*^2 patients had missing data for socioeconomic class

^*^One patient had missing data for closure of skin surgical incision

^*^Of those who received vertical tracheal incisions, maturation sutures were only placed for 11 patients (under 18 years who received it). Other admission diagnosis included; Abdominal trauma, cut throat injury, lingual cancer, and temporomandibular joint ankylosis. *OMF* oromaxillofacial, *ICU* intensive care unit, *HDU* high dependency unit, *ENT* ear, nose and throat

### Indications for tracheostomy

Multiple indications for tracheostomy were noted in 69% of patients. The most common indications were; pulmonary toilet (58%), anticipated prolonged intubation (42%), upper airway obstruction (36%) and airway protection (28%). There were no significant disparities in all indications across hospitals except for airway protection which was more commonly encountered in patients at MNRH (40%) as compared MRRH (16%), *p* = 0.008 Table 2.

**Table 2:** Indications for tracheostomy, overall and by hospital stratification

Indication for tracheostomy	Overall, *N* = 100, n (%)	MNRH, *N* = 50, n (%)	MRRH, *N* = 50, n (%)	*p*-value
Pulmonary toilet	58 (58.0)	31 (62.0)	27 (54.0)	0.418
Anticipated prolonged intubation	42 (42.0)	18 (36.0)	24 (48.0)	0.224
Upper airway obstruction	36 (36.0)	20 (40.0)	16 (32.0)	0.405
Airway protection	28 (28.0)	20 (40.0)	8 (16.0)	0.008
Anticipated difficult intubation	9 (9.0)	3 (6.0)	6 (12.0)	0.295
Adjunct to head and neck surgery	4 (4.0)	3 (6.0)	1 (2.0)	0.119
Failed intubation	2 (2.0)	1 (2.0)	1 (2.0)	1.000
Failed extubation	1 (1.0)	0 (0.0)	1 (2.0)	0.591

N = total number of patients, n = number of patients who had particular indications, some had multiple indications

Table 3 shows that upper-airway obstruction was the commonest indication among males (93.7%) while airway protection was the commonest among females (62.5%) at MRRH, and this difference was statistically significant, *p* < 0.05. There were also no major disparities in indications of tracheostomy between adult and child patients in MRRH. Overall, at MNRH, no significant disparities were noted in indications for tracheostomy across gender and age categories.

**Table 3:** Indications of tracheostomy stratified by age and gender and their variation between hospitals

Variable	Indication							
	Pulmonary toilet: *N* = 58		Anticipated prolonged intubation: *N* = 42		Upper airway obstruction: *N* = 36		Airway protection: *N* = 28	
	MRRH *N* = 27, n (%)	MNRH *N* = 31, n (%)	MRRH *N* = 24, n (%)	MNRH *N* = 18, n (%)	MRRH *N* = 16, n (%)	MNRH *N* = 20, n (%)	MRRH *N* = 8, n (%)	MNRH *N* = 20, n (%)
Gender- specific								
Male	17 (63.0)	20 (64.5)	15 (62.5)	11(61.1)	15 (93.7)	14 (70.0)	3 (37.5)	14 (70.0)
Female	10 (37.0)	11 (35.5)	9 (37.5)	7 (38.9)	1 (6.3)	6 (30.0)	5 (62.5)	6 (30.0)
* p*-value	0.054	0.777	0.075	0.584	**0.029**	0.626	**0.010**	0.626
Age category								
Pediatric (< 18)	3 (11.1)	8 (25.8)	1 (4.2)	4 (22.2)	2 (12.5)	3 (15.0)	1 (12.5)	3 (15.0)
Adults (≥ 18)	24 (88.9)	23 (74.2)	23 (95.8)	14 (77.8)	14 (87.5)	17 (85.0)	7 (87.5)	17 (85.0)
* p*-value	0.777	0.407	0.187	0.977	0.686	0.329	0.797	0.329

### Tracheostomy-related complications

Overall, 53 patients developed complications, giving an incidence of early tracheostomy-related complications of 53% (95% CI: 43.0 – 62.7). No significant difference in incidence was noted between MRRH and MNRH, *p* = 0.841.

A total of 76 complication events (38 in MRRH and 38 in MNRH) occurred among the 53 patients. Thirty-five patients developed one complication each while 18 patients developed multiple complications of whom 14 developed 2 complications and 4 developed more than two complications each. The commonest complications were; tube obstruction (52.6%), inadvertent decannulation (17.1%) and bleeding (11.8%) Table 4.

**Table 4:** Incidences of specific early complications, overall and by hospital stratification

Complication	Overall, *N* = 76, n (%)	MNRH, *N* = 38, n (%)	MRRH, *N* = 38, n (%)	*p*-value
Tube obstruction	40 (52.6)	20 (52.6)	20 (52.6)	1.000
Inadvertent decannulation	13 (17.1)	10 (26.3)	3 (7.9)	**0.037**
Bleeding	9 (11.8)	4 (10.5)	5 (13.2)	0.727
Stomal site infection	5 (6.6)	3 (7.9)	2 (5.3)	0.646
Subcutaneous emphysema	3 (3.9)	0 (0.0)	3 (7.9)	0.079
Dysphagia	3 (3.9)	1 (2.6)	2 (5.3)	0.558
Stomal granulation	2 (2.6)	0 (0.0)	2 (5.3)	0.153
Aspiration	1 (1.3)	0 (0.0)	1 (2.6)	0.315

There was no significant difference in the distribution of the common tracheostomy-related complications between MRRH and MNRH except for inadvertent decannulation which was commonest at MNRH (26.3%) as compared to 7.9% at MNRH (*p* = 0.037).

### Factors associated with development of early tracheostomy-related complications

Table 5 shows the results of univariate and multivariate analysis for factors associated with tracheostomy related complications.

**Table 5:** Results of univariate and multivariate analysis for factors associated with early tracheostomy-related complications

Variable	Univariate analysis			Multivariate analysis	
	Complication, n (%)	No Complication, n (%)	Unadjusted RR (95% CI)	Adjusted RR (95% CI)	*p*-value
Hospital					
Mbarara	26 (52.0)	24 (48.0)	1.0		
Mulago	27 (54.0)	23 (46.0)	1.1 (0.72 – 1.50)		
Gender					
Female	12 (40.0)	18 (60.0)	1.0	1.0	0.132
Male	41 (58.6)	29 (41.4)	1.5 (0.91 – 2.36)	1.4 (0.91 – 2.04)	
Age category (Years)					
Adults (≥ 18)	43 (51.2))	41 (48.8)	1.0	1.0	**0.048**
Pediatric (< 18)	10 (62.5)	6 (37.5)	1.2 (0.79 – 1.88)	1.2 (1.00 – 1.47)	
Post-operative feeding mode					
Nasogastric tube	31 (45.6)	37 (54.4)	1.0		
Oral feeding	22 (68.7)	10 (31.3)	**1.5 (1.06 – 2.13)**		
Admission diagnosis					
Upper airway obstruction	23 (67.7)	11 (32.3)	1.0	1.0	
TBI/stroke	28 (46.7)	32 (53.3)	0.7 (0.48 – 0.98)	0.8 (0.57 – 1.44)	0.234
Others	02 (33.3)	04 (66.7)	0.5 (0.15 – 1.56)	0.5 (0.15 – 1.84)	0.317
Post-operative ventilation mode					
Spontaneous	19 (41.3)	27 (58.7)	1.0		
Ventilator assisted	34 (63.0)	20 (37.0)	**1.5 (1.02 – 2.27)**		
Site for surgery					
Wards (ENT/OMF)	7 (50.0)	7 (50.0)	1.0	1.0	
Emergency/casualty	4 (57.2)	3 (42.8)	1.4 (0.49 – 2.62)	1.2 (0.55 – 2.82)	0.609
ICU/HDU	25 (46.3)	29 (53.7)	0.9 (0.51 – 1.68)	1.6 (0.94 – 2.79)	0.083
Operating theatre	17 (68.0)	8 (32.0)	1.4 (0.75 – 2.45)	1.5 (0.88 – 2.64)	0.127
Indication for tracheostomy					
Pulmonary toilet	29 (50.0)	29 (50.0)	1.0	1.0	
Anticipated prolonged intubation	16 (38.1)	26 (61.9)	**1.7 (1.08 – 2.58)**	1.8 (1.19 – 2.76)	**0.005**
Airway protection	16 (57.1)	12 (42.9)	1.1 (0.75 – 1.64)	1.4 (0.79 – 1.64)	0.605
Anticipated difficult intubation	5 (55.6)	4 (44.4)	1.1 (0.57 – 1.95)	1.3 (0.57 – 1.90)	0.872
Upper airway obstruction	24 (66.7)	12 (33.3)	**1.5 (1.03 – 2.09)**	1.5 (1.03 – 2.09)	0.062
Tracheostomy type					
Elective	28 (49.1)	29 (50.1)	1.0		
Emergency	25 (58.0)	18 (42.0)	1.2 (0.82 – 1.70)		
Type of tracheal incision					
T-Incision	17 (43.6)	22 (56.4)	1.0	1.0	
Bjork flap	8 (88.9)	1 (11.1)	**2.0 (1.33 – 3.11)**	1.6 (1.09 – 2.43)	**0.016**
Vertical	26 (54.2)	22 (45.8)	1.2 (0.79 – 1.93)	1.5 (1.02 – 2.27)	**0.037**
Horizontal	2 (50.0)	2 (50.0)	1.1 (0.40 – 3.25)	1.6 (0.83 – 2.99)	0.165
Cuffed tracheostomy tube					
Yes	45 (50.6)	44 (49.4)	1.0		
No	8 (72.7)	3 (27.3)	1.4 (0.94 – 2.18)		
Peri-operative antibiotics					
Yes	27 (45.0)	33 (55.0)	1.0		
No	26 (65.0)	14 (35.0)	**1.4 (1.00 – 2.07)**		

*TBI* Traumatic brain injury, *OMF* oromaxillofacial, *ICU* intensive care unit, *HDU* high dependency unit, *ENT* ear, nose and throat

In univariate analysis, the statistically significant factors were; Oral feeding post-operatively, assisted post-operative ventilation, having an indication of anticipated prolonged intubation or upper airway obstruction, use of Bjork flap tracheal incision and peri-operative antibiotic use. In addition to these factors, admission diagnosis, age, gender, and the site for surgery were included in the final model and adjusted for.

In multivariate analysis, the only factors with a significant independent association with early tracheostomy-related complications were; being younger than 18 years, having an indication for tracheostomy of anticipated prolonged intubation, and having either a Bjork flap or a vertical tracheal incision.

Patients having anticipated prolonged intubation as the indication for tracheostomy was associated with a 1.8 times risk of developing early complications compared to patients with pulmonary toilet as their indication (RR = 1.8, 95%CI: 1.19 – 2.76, *p* = 0.005). Having a Bjork flap tracheal incision was associated with a 1.6 times risk of developing early complications (RR = 1.6, 95%CI: 1.09 – 2.43, *p* = 0.016) while those with vertical incision had a 1.5 times risk compared to those that received a T-incision (RR = 1.53, 95%CI: 1.02 – 2.27, *p* = 0.037). Being younger than 18 years was associated with a 1.2 times risk of early complications as compared to being 18 years and above (RR = 1.22, 95%CI2: 1.00 – 1.47, *p* = 0.048).

## Discussion

### Indications for tracheostomy

In this study, we observed that a considerable proportion (69%) of patients had more than one tracheostomy indication. This finding was probably because most (54%) of study participants were very ill ICU and HDU patients who are likely to have multiple challenges for example; reduced consciousness hence unable to protect their airways and clear airway secretions yet at the same time require prolonged intubation, all of which are recognized tracheostomy indications. This study found a comparable distribution of indications at the two study sites probably because of the similarity in patients’ sociodemographic characteristics as shown in Table 1, or disease epidemiology and risk for trauma given that they are both urban-based hospitals. We also found pulmonary toilet as the commonest indication which is at variance with what was previously reported in various African studies that found upper airway obstruction as the commonest indication [6, 19–21] while others reported anticipated prolonged intubation as the commonest at 55.2% and 95% in Rwanda and India respectively [22, 23]. The discrepancy may have been because comparison studies that found upper airway obstruction as commonest differed from ours regarding to admission diagnoses. They reported significant contributions from aero-digestive infections and impacted airway foreign bodies among children, and aerodigestive trauma and tumors especially among their adult patients all of which contributed to the increased incidence of upper air-way obstruction. Our study also had few pediatric patients (16%) and with no patients with aero-digestive infections or airway foreign bodies. Overall, the commonest form of trauma we found was traumatic brain injury rather than aerodigestive trauma reported in other studies. In the same vein, the Indian study had 59% and 21.3% of their patients admitted due to severe organophosphate poisoning and snake bites respectively while in Rwanda, all their patients were recruited from ICU and had history of severe trauma, all of whom were likely to require prolonged periods of intubation for airway support. The present study however did not find similar indications and the trauma severity was not assessed.

### Early tracheostomy-related complications

The 53% incidence of early tracheostomy-related complications documented in this study, although in alignment with the global estimated range of 6 to 66%, is higher than the 21.5% reported in Tanzania [7]. Similarly, this incidence is higher than what has been reported in previous studies from comparable settings such as Nigeria where this rate ranged between 10.3% and 21% [6, 20] and India (29.8%) [24]. Lower complication rates (15%) have been documented in high income settings such as Finland [25]. Notably most of these studies were retrospective and considered all post-procedure complications and not only early complications. The high incidence in our study could be attributed to the cadre of surgeons performing the procedure in our case who were mainly residents in training unlike similar studies where mainly specialist otolaryngologists with arguably better skills and longer experience performed the procedures [25]. The observational nature of this study too could have triggered an increased intentional surveillance for complications resulting in the documentation of even more subtle episodes of tube obstruction hence providing a higher incidence as also seen in Kenya [14]. The quality of care given to patients could also have contributed to the high incidence of complications in the current study. The effectiveness of the five drivers of tracheostomy care improvement including; use of multidisciplinary tracheostomy care teams, standardized tracheostomy care protocols or pathways, staff education, patient and family involvement, and collection of patient-level data [4, 26] as recommended by the global tracheostomy collaborative was recently demonstrated in UK [27] but in the current study setting, only staff education, and patient and family involvement (2 of 5) are being implemented.

### Incidence of specific early complications

Our study reported tracheostomy tube obstruction as the commonest early complication (52.6%) which agrees with another study in similar settings that reported it at 80.3% in Kenya [15]. Our finding could be due to the fact that majority (72%) of tubes used had no disposable internal cannula in addition to absence of heat and moisture exchangers. The internal cannula is useful in trapping mucus secretions, which prevents clogging of the larger tube. It also makes it easy for patients and their attendants to participate in tube care by removing, cleaning and replacing the cannula on a regular basis. On the other hand, a heat and moisture exchanger is a filter fitted onto the tracheostomy tube to keep the airway humidified. This helps to thin out secretions, making them easy to either expectorate or suction out. In addition, as similarly highlighted by the Nyansikera and Kirui study, low staffing could also have made it harder to achieve the minimum of 3 times per day tracheostomy tube suctioning as recommended [28] to prevent this complication. In the absence of these vital resources, training of health workers and patient attendants on the proper procedure for tube suctioning can greatly reduce the incidence of tube obstruction. Life-threatening complications like tracheo-innominate fistula, pneumothorax and pneumomediastinum were not reported in this study and no mortality occurred as a result of tracheostomy.

### Factors associated with development of early tracheostomy-related complications

Bearing in mind tracheostomy tube obstruction as the commonest complication in this study, our finding that anticipated prolonged intubation as an indication was associated with increased risk of early complications compared to pulmonary toilet may be because patients who were operated for this indication were more likely to have had a significantly severe illness. Such patients would still be too sick to self-care for their tracheostomy tubes even in the post-procedure period and this may be compounded by their inability to communicate a need to be suctioned or to have the tube cleaned or changed in addition to a higher possibility of a poor cough reflex. All these may have increased chances of retention of copious airway secretions hence a higher risk of tube obstruction. Such patients are also more likely to require assisted bed turning and bathing compared to more stable patients and this increases chances of inadvertent tube decannulation, which was in fact the second most common complication (17.1%) in this study.

Our finding that a patient being younger than 18 years was associated with increased risk of early complications was not surprising since this association had previously been reported by a study in a similar setting that found a higher complication rate among children below 10 years as compared to older patients [7]. This is in agreement with our study in which 10 out of the 16 children enrolled were below 10 years of age. This age group could be more prone to complications because they require smaller caliber tracheostomy tubes which are more prone to obstruction in addition to their anatomical features such as a shorter neck, more pliable laryngeal structures and more prominent subcutaneous fat. Pediatric tracheostomy requires procedural modifications if complications are to be minimized, for example, neck hyperextension may pull mediastinal structures in to the neck thus increasing chances of damage to lung apices and resultant emphysema, pneumothorax and pneumomediastinum. Also, children are less able to independently care for their tracheostomy sites and are still less likely to communicate the need to be suctioned which increases chances of tube obstruction as compared to adults.

The Bjork flap is an inferiorly based tracheal flap through the 2^nd^, 3^rd^, and 4^th^ tracheal rings which is fixed to the skin to stabilize the tracheal lumen. Although it has a risk for tracheal stenosis, it has advantages over traditional incisional and excisional window procedures including; reduced risk of false cannulation especially during emergency recannulation following accidental tube dislodgement, early stomal maturation and ease of stomal care by assistants and family members [29–31]. These make it the preferred tracheal incision in our setting for patients who require tracheostomy for longer durations such as those with head and neck tumors. However, its indications may vary, as it was preferred for patients with prolonged mechanical ventilation and elevated body mass index while window procedures were used in head and neck cancer patients in USA [32].

Our finding that Bjork flap and vertical tracheal incisions were associated with increased risk of early complications may have been due to other factors surrounding their indication for tracheostomy. These two tracheal incisions are in themselves indicated in patients with an already increased risk for tracheostomy-related complications. For example, the Bjork flap is preferred for patients with head and neck cancer who already have increased risk for complications such as aspiration and tube displacement. The vertical incision is preferred for emergency tracheostomy and for children because it is faster and poses a low risk of severing paratracheal structures. However, in emergency situations, the risk complications such as bleeding, stomal site infection and surgical emphysema are increased [33, 34]. It is important to note that in the current study, all Bjork flaps were performed in head and neck cancer patients and all vertical tracheal incisions were performed in emergency procedures (43% of all procedures).

Despite earlier literature from a high income setting reporting up to 73% patients to have made tracheostomy-related post-discharge emergency room visits [35], it is still unknown as to how tracheostomy patients in our setting transition from hospital care to community care and how they fair post-discharge. Considering the high in-hospital complication rate in the current study, giving extra attention to their discharge preparedness and community integration would contribute greatly to their tracheostomy care while in the community.

The strength of our study is that it gives information on tracheostomy-related complications in low resource settings. The exclusion of patients with a known underlying risk of bleeding as per our exclusion criteria could have resulted in a possible under-estimation of the incidence of bleeding as a complication and the overall complication rate in the study population. However, we had no patient who was excluded based on this criterion, hence eliminating the impact of this anticipated limitation. The small sample size may have limited our ability to establish true associations between complications and the risk factors identified in addition to the small number of pediatric patients in this study which may affect the generalizability of our study results to the pediatric age group.

## Conclusion and recommendation

Pulmonary toilet is the commonest tracheostomy indication at major hospitals in Uganda. The incidence of early tracheostomy-related complications is high and majorly related to post-procedure tracheostomy tube management. Having anticipated prolonged intubation as an indication for tracheostomy, having had a Bjork flap or vertical tracheal incisions and being a child was associated with increased risk of complications. Emphasis on multidisciplinary team care, standardization of tracheostomy care protocols, and continuous collection of patient data as well as paying attention to patient quality of life factors such as early return to oral feeding, ambulation and normal speech may have great potential for improved quality of tracheostomy care in low resource settings.

## Data Availability

The datasets used and/or analyzed during the current study are available from the corresponding author on reasonable request.

## Abbreviations

COVID-19: Corona Virus Disease—2019
ENT: Ear, Nose and Throat
HDU: High Dependency Unit
ICU: Intensive Care Unit
MNRH: Mulago National Referral Hospital
MRRH: Mbarara Regional Referral Hospital
MUST REC: Mbarara University of Science and Technology Research Ethics Committee
OMF: Oromaxillofacial
TBI: Traumatic Brain Injury
